# Multi-junction cascaded vertical-cavity surface-emitting laser with a high power conversion efficiency of 74%

**DOI:** 10.1038/s41377-024-01403-7

**Published:** 2024-02-28

**Authors:** Yao Xiao, Jun Wang, Heng Liu, Pei Miao, Yudan Gou, Zhicheng Zhang, Guoliang Deng, Shouhuan Zhou

**Affiliations:** 1 College of Electronics and Information Engineering, Sichuan University, Chengdu, 610064 China; 2 Suzhou Everbright Photonics Co., Ltd, Suzhou, 215163 China

**Keywords:** Semiconductor lasers, Green photonics

## Abstract

High electro-optical conversion efficiency is one of the most distinctive features of semiconductor lasers as compared to other types of lasers. Its further increase remains a significant objective. Further enhancing the efficiency of edge-emitting lasers (EEL), which represent the highest efficiency among semiconductor lasers at present, is challenging. The efficiency of vertical cavity surface emitting lasers (VCSELs) has always been relatively low compared to EEL. This paper, combining modeling with experiments, demonstrates the potential of multi-junction cascaded VCSELs to achieve high efficiency beyond that of EELs, our simulations show, that a 20-junction VCSEL can achieve an efficiency of more than 88% at room temperature. We fabricated VCSEL devices with different numbers of junctions and compared their energy efficiency. 15-junction VCSELs achieved a maximum efficiency of 74% at room temperature under nanosecond driving current, the corresponding differential quantum efficiency exceeds 1100%, being the largest electro-optical conversion efficiency and differential quantum efficiency reported until now for VCSELs.

## Abstract (web summary)

We have theoretically and experimentally proven the potential of multi-junction VCSELs to achieve high electro-optical conversion efficiency, providing insights for the further development and application of high efficiency semiconductor lasers.

## Graphical abstract

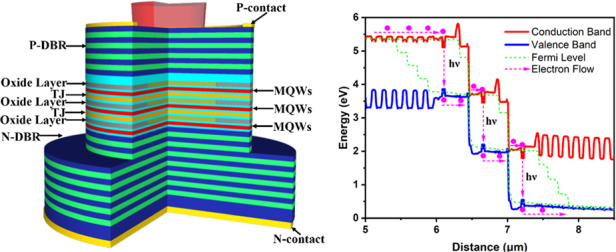

## Introduction

Since the advent of semiconductor laser in 1962, they have undergone rapid advancements^1–3^. Compared to other types of lasers, such as gas lasers, solid-state lasers, and fiber lasers^4,5^, semiconductor lasers have numerous advantages, with the most notable being their ability to achieve extremely high electro-optical conversion efficiency. The pursuit of ultra-high efficiency in semiconductor lasers remains a significant objective in photonics and laser physics. Since the inception of edge-emitting semiconductor lasers (EEL), The record for the highest power conversion efficiency (PCE) has been continuously broken^6–9^. Thus far, the highest efficiency record for EEL was set in 2006, achieving a conversion efficiency of 85% at −50 °C^10^. Subsequently, in the following year, EEL achieved a maximum PCE of 76% at room temperature^11^. However, no new records have emerged in the past fifteen years. Meanwhile, these PCE records have always represented the top records for all semiconductor lasers. Compared to EEL, The PCE of VCSELs has been increasing very slowly and there is a significant disparity^12,13^. After reporting a maximum PCE of 62% for single-junction VCSELs in 2009^14^, there has been no breakthrough progress for over a decade. It’s commonly held that for VCSELs, which are microcavity lasers, attaining high power conversion efficiency record has perennially been an elusive goal in photonics.

Therefore, early applications of VCSELs were mainly focused on consumer electronics requiring small-volume, low-power light sources (such as mice and printers) and short-distance optical communication in data centers^15^. In the past decade, with the development of intelligent technology, VCSELs have become the core light source for smart sensing systems and have been widely and maturely applied in facial recognition and short-range sensing systems, achieving significant success^16,17^. However, in recent years, with the rapid development of more advanced artificial intelligence(AI) technologies, VCSELs face both tremendous prospects and challenges in the fields of sensing^18^, communication^19^, atomic clock^20^, optical or quantum computing^21,22^, topological laser^23^, and medical examination^24^.

With the rapid advancement of long-distance sensing technology in autonomous driving, advanced AI computations like ChatGPT demanding high data capacity and speed, and the swift growth of intelligent and quantum technology applications such as deep learning based on VCSELs^25,26^, these technological domains inevitably face a critical shared challenge: the issue of energy consumption. Whether it’s the battery consumption of mobile terminals or the energy consumption of data centers, VCSELs, as light sources, constitute a significant part of the energy drain. Especially with the rapid development of AI computations, the energy consumption requirements for data centers will further increase, expected to grow by an order of magnitude by 2030^27^. It is significant to note that researching ultra-high-efficiency VCSELs plays a crucial role in promoting the development of the future intelligent era.

The primary reason for the consistently low PCE of VCSELs is that, being a microcavity laser, it has an extremely small cavity volume, leading to a significant reduction in its round-trip gain. A low gain volume results in a high threshold current. To achieve the goal of a low threshold, VCSEL structural designs typically incorporate high reflectivity mirrors formed by distributed Bragg reflectors (DBRs) grown at both the top and bottom. However, this design results in a significant increase in resistance, subsequently leading to Joule heating. In addition, doping within the DBR can cause carrier absorption losses. These two factors collectively limit the efficiency of VCSELs. Therefore, in 2009, Takaki et al. reported their use of a dual-cavity internal contact structure^14^, featuring an undoped bottom semiconductor DBR and a dielectric top DBR, to maximize the reduction in overlap volume between electrical and optical paths, optical losses, and series resistance, thereby achieving a high PCE of up to 62% for single-junction VCSELs at room temperature under continuous-wave(CW) operation. But, this lateral current injection structure is very challenging to implement in two-dimensional array fabrication. In the past decade, the electro-optical conversion efficiency of VCSELs has not seen significant improvement. The electro-optical conversion efficiency of single-junction VCSELs has reached a bottleneck.

However, it’s worth noting that as early as 1984, Iga proposed an innovative method^28^, which involved using reverse tunneling junctions to achieve cascading of the active region, thereby increasing the gain volume. This design strategy allows carriers to undergo multiple stimulated emission processes, enabling the device to not only exhibit a high differential quantum efficiency but also maintain a lower threshold current. Thus, by cascading additional active regions, multi-junction VCSELs can realize a substantial gain increase, but the serial resistance and intrinsic losses don’t multiply in the same manner, resulting in a boost in PCE. Early research on multi-junction VCSELs primarily focused on enhancing differential quantum efficiency and output power by cascading more active regions. In 1999, T. Knodl et al.^29^ first achieved a 3-junction 980 nm VCSEL operating at room temperature under pulsed driving, with a differential quantum efficiency of 8%. In 2001, T. Knodl et al. achieved a maximum output power of ~9 mW for a 3-junction VCSEL operating at room temperature in CW mode, with a differential quantum efficiency of 130%^30^. At the same time, they also conducted theoretical research on multi-junction VCSELs, primarily focusing on the threshold gain and threshold current, differential quantum efficiency, voltage, and other characteristics of multi-junction VCSELs. By around 2017, VCSELs had achieved remarkable accomplishments in the field of sensing, Multi-junction VCSELs are the ideal choice for LIDAR applications^31,32^. The performance of multi-junction VCSELs has also seen significant improvements. In 2021, we reported that the 940 nm 3-junction VCSEL achieved a PCE of 61.3% under continuous operation at room temperature^33^. However, multi-junction cascaded EEL cannot achieve efficiency improvement. They are merely a vertical stacking of multiple independent lasers. Although to date, most research on multi-junction VCSELs still focuses on their high differential quantum efficiency and high output power characteristics, but compared to edge-emitting semiconductor lasers, the most significant potential advantage of multi-junction VCSELs should be their remarkable efficiency improvement. Existing multi-junction VCSEL studies have focused on highlighting the advantages of high slope efficiency, achieving higher power^34–37^. However, reports on systematically studying the efficiency of multi-junction VCSELs by combining theoretical simulations with experiments are still relatively scarce. Our paper explores the transition from single to multi-junction VCSELs, analyzing their scaling properties and efficiency advantages through both theoretical and experimental approaches, thereby achieving the highest efficiency presently reported for VCSELs.

This paper demonstrates the advantages of multi-junction cascaded vertical-cavity surface-emitting lasers (multi-junction VCSELs) in providing ultra-high efficiency laser output, and It is expected to further surpass the PCE of EEL at room temperature and elevate the PCE of semiconductor lasers at room temperature to a higher level. We have undertaken simulation and design studies on multi-junction VCSELs. The results from numerical simulations suggest that a 20-junction VCSEL can attain an energy conversion efficiency exceeding 88% at room temperature. This PCE value has already surpassed the highest PCE value reported under low-temperature operating conditions in experimental studies of EEL. At the same time, we manufactured VCSEL devices with varying junction VCSEL and carried out a comparison regarding their energy dissipation ratios. Experimentally, we have shown that the 15-junction VCSEL can reach an energy conversion efficiency as high as 74% at room temperature under nanosecond pulse driving. corresponding differential quantum efficiency exceeds 1100%. To the best of the author’s knowledge, this is the highest efficiency device in VCSELs to date. Meanwhile, this differential quantum efficiency is the world records in the semiconductor laser field to date.

## Results

### Simulation results

In order to understand scaling properties of multi-junction VCSELs, it is important to derive a relationship between threshold gain and the number of active layers N, as well as threshold current density and differential quantum efficiency. The specific theory of the scaling behavior of Multi-junction VCSELs is presented in the first part of the supplementary materials. Parameters for the numerical simulation are listed in Supplementary Table 1.

The threshold condition of the VCSEL^38^:

1 $$\varGamma {{\rm{g}}}_{th}=\alpha +\frac{1}{{l}_{c}}\,\mathrm{ln}\,\frac{1}{\sqrt{{R}_{b}{R}_{t}}}$$

In a VCSEL the scaling behavior of multi-junction VCSELs with respect to a conventional single-junction VCSEL can be described as follows. The detailed derivation process can be found in the supplementary materials. The threshold gain:

2 $${g}_{n}=\frac{{g}_{1}}{N}+\frac{{\alpha }_{i}\varDelta {l}_{c}}{\varGamma {l}_{a1}N}-\frac{\mathrm{ln}\,\sqrt{\zeta }}{\varGamma {l}_{a1}N}$$

and the threshold current density:

3 $${J}_{N}={J}_{1}\exp \left[\frac{1}{{g}_{0}N}\left({g}_{1}(1-N)+\frac{{\alpha }_{i}\varDelta {l}_{c}}{\varGamma {l}_{a}}-\frac{\mathrm{ln}\,\sqrt{\zeta }}{\varGamma {l}_{a}}\right)\right]$$

**Fig. 1:**
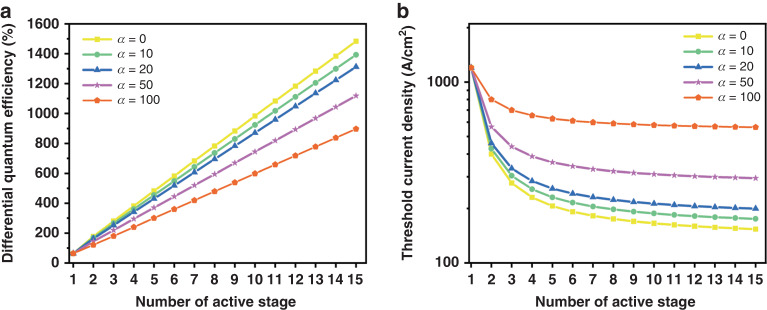
The scaling properties of differential quantum efficiency and threshold gain for VCSELs with different numbers of junctions. **a** The relationship between differential quantum efficiency with the number of junctions. α_i_ represents the internal loss of the VCSELs. As the number of junctions increases, the differential quantum efficiency exceeds 1000%. **b** The relationship between threshold current density with the number of junctions. α_i_ represents the internal loss of the VCSELs. The ordinate is a logarithmic coordinate. As the number of junctions increases, the threshold current density gradually decreases

From Eq. (3), it can be observed that J decreases as N increases. This indicates the scalability of the threshold current density with respect to the value of a single-junction. The relationship between threshold current with the number of junctions is shown in the following Fig. 1b.

The differential quantum efficiency reveals an almost linear increase with respect to *N*, namely:

4 $${\eta }_{dN}=\frac{N{\eta }_{d1}\,\mathrm{ln}\,\sqrt{\zeta {R}_{{\rm{b}}}{R}_{t}}}{\mathrm{ln}\sqrt{{R}_{{\rm{b}}}{R}_{t}+{\eta }_{d1}(\mathrm{ln}\,\sqrt{\zeta }-\alpha \varDelta L)/{\eta }_{i1}}}$$

The relationship between differential quantum efficiency with the number of junctions is shown in the following Fig. 1a. When the internal loss in the cavity is minimal, the linear correlation between differential quantum efficiency and N is more evident. As the number of junctions increases, the differential quantum efficiency exceeds 1000%. As of now, the highest differential quantum efficiency documented in experiments is the 620% realized through an 8-junction VCSEL, as we reported in 2022^39^. Therefore, based on this characteristic, current extensive research on multi-junction VCSELs focuses on enhancing the device’s differential quantum efficiency through multi-junction cascading, thereby achieving higher output power at the same driving current^34,35,39^. Multi-junction VCSELs, which offer high power and surface emission with a low-temperature wavelength shift coefficient, have consequently emerged as one of the optimal laser sources for all-solid-state LiDAR in autonomous intelligent terminals, and have been extensively utilized in recent years. However, few studies have concentrated on the scaling properties and experimental research of multi-junction VCSELs in terms of electro-optical conversion efficiency. Our theoretical research here has revealed significant potential in this aspect of multi-junction VCSELs, and we have experimentally confirmed improvements in their electro-optical conversion efficiency.

Combining the threshold current and differential quantum efficiency, we can derive the following equation for PCE:

5 $$PCE=\frac{{P}_{{\rm{laser}}}}{VI}=\frac{(I-{I}_{th})slope}{I{V}_{on}+IR},\,slope={\eta }_{dN}\frac{{\rm{h}}\nu }{e}$$

**Fig. 2:**
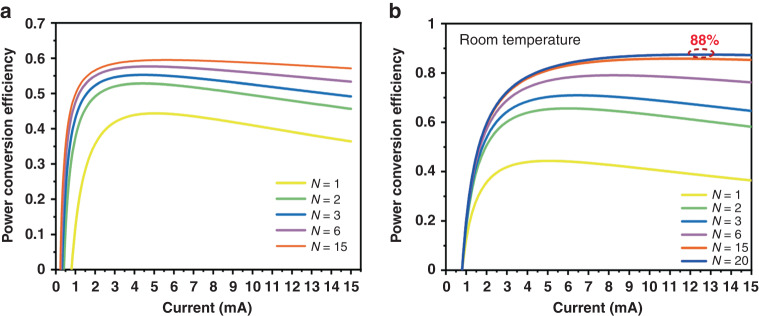
PCE numerical simulation of VCSEL with different junction numbers. **a** Output reflectivity remains constant, the threshold significantly decreases with the increase in the number of junctions, and the PCE rises from 45% for a single-junction VCSEL to 60% for a 15-junction VCSEL. **b** Threshold remains constant, the PCE of VCSELs can be increased from 48 to 88%

To delve deeper into this issue, we conducted numerical simulations based on Eq. (5). The detailed derivation process can be found in the supplementary materials. The parameters for the simulation are listed in Supplementary Table 1. This simulation was based on experimental data of a single-junction VCSEL operating continuously at room temperature. Based on Equations 14 and 15 in the supplementary materials, we simulated the scaling characteristics of the power conversion efficiency of multi-junction VCSELs under two conditions: maintaining a constant reflectivity of the top output mirror and keeping the threshold gain unchanged. It’s worth noting that during the simulation, we did not consider the impact of reduced internal quantum efficiency due to excessive thermal power at low currents as the number of junctions increased. The simulation results are shown in Fig. 2. Figure 2a clearly indicates that as the output reflectivity remains constant, the threshold significantly decreases with the increase in the number of junctions, and the PCE rises from 45% for a single-junction VCSEL to 60% for a 15-junction VCSEL. However, even with an increased number of junctions, the maximum PCE of VCSEL will not exceed 65%. If the threshold remains constant, to increase the number of junctions, we need to appropriately reduce the output reflectivity, decreasing from 99.5% in a single-junction VCSELs to 75% in a 15-junction VCSELs. As can be seen from Fig. 2b, with the number of junctions in VCSELs increases, the PCE of VCSELs has a significant improvement. the PCE of VCSELs can be increased from 48 to 88%, achieving around 80% improvement. This PCE value has already surpassed the highest PCE value reported under low-temperature operating conditions in experimental studies of EEL. To achieve an 88% electro-optical conversion efficiency in a 20-junction VCSEL, it is imperative to ensure that each pn-junction exhibits quantum well structures of high-quality growth. In addition, optimization of the device’s output reflectivity and doping design is crucial. Concurrently, it is essential to maintain the series resistance at a comparatively low level to ensure efficient performance.

Clearly, the primary advantage of multi-junction VCSELs is their ability to significantly enhance PCE at room temperature by increasing the number of junctions, effectively addressing a major technical bottleneck of VCSELs compared to edge-emitting lasers. This performance improvement offers a potent solution to the energy consumption issues of VCSELs in their widespread future applications. Considering this, we plan to further explore the potential applications of multi-junction VCSELs in the field of communication in the future. It’s worth noting that T. Knodl and colleagues also pointed out in their theoretical analysis that the modulation rate of multi-junction VCSELs is expected to see a significant increase^40^. This is because the series connection of multiple PN junctions can effectively reduce the overall capacitance of the resonant cavity. The increased gain volume allows us to maintain a low threshold while reducing the top reflectivity, thereby decreasing the photon lifetime and enhancing the modulation bandwidth^41^.

### Experiment results

**Fig. 3:**
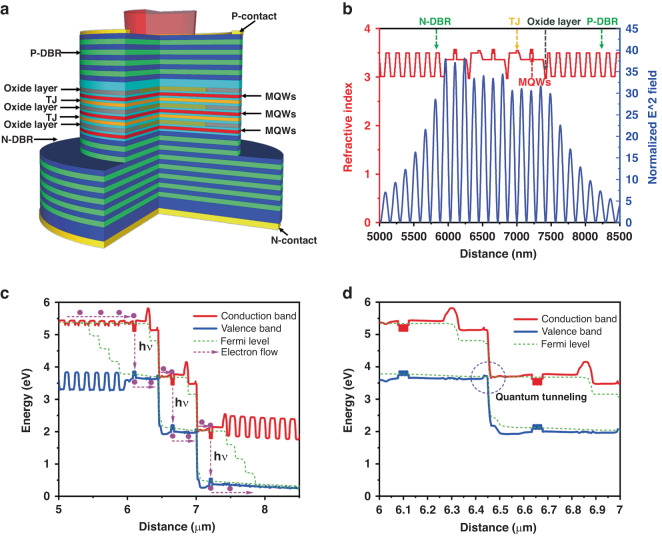
Schematic diagram of device structure. **a** Structural schematic diagram, including N-type Distributed Bragg Reflector (N-DBR), P-type Distributed Bragg Reflector (P-DBR), multiple quantum wells (MQWs), oxidation layer, tunnel junction (TJ). **b** Refractive index distribution, Standing wave light field distribution, **c** active region band structure of 3-junction VCSEL under bias. By cascading multiple active regions through tunnel junctions, a mechanism for multiple electron transitions was realized. **d** Tunnel junction band structure under bias

We fabricated samples of 1-junction, 6-junction, 8-junction, and 15-junction VCSELs. To provide a more intuitive display of the design features of the multi-junction structure, Fig. 3 illustrates structural schematic diagram, the refractive index distribution, standing wave light field distribution, and the energy band structure of the active region for a three-junction VCSEL. These simulation results were obtained using the Crosslight software. For VCSELs with a higher number of junctions, their structure can be viewed as multiple repeated superpositions of the active region. As shown in Fig. 3b, The quantum well was designed at the antinode of the standing wave light field to enhance the coupling efficiency of carriers and photons, thereby increasing the gain. The oxidation layer was designed near the second node of the standing wave light field close to the quantum well. The highly doped tunnel junction was located near the third node of the standing wave light field above the quantum well, which significantly reduces the absorption loss of free carriers. Figure 3c illustrated the band structure of the active region of the 3-junction VCSELs under bias. The pink dashed line outlined the microscopic transport process of electrons: Electrons flowed from the conduction band of the N-type DBR to the first multiple quantum wells (MQWs), where carrier recombination occurred, emitting photons, and in the process, electron transitioned from the conduction band to the valence band. When electrons moved to the highly doped tunnel junction (TJ), quantum tunneling effects occurred, as shown in Fig. 3d, allowing electrons to tunnel from the valence band to the conduction band and they continued to flow to the next quantum well, where carrier recombination and photon emission occurred again. By cascading multiple active regions through tunnel junctions, a mechanism for multiple electron transitions was realized, thereby enhancing the differential quantum efficiency and increasing the gain volume.

**Fig. 4:**
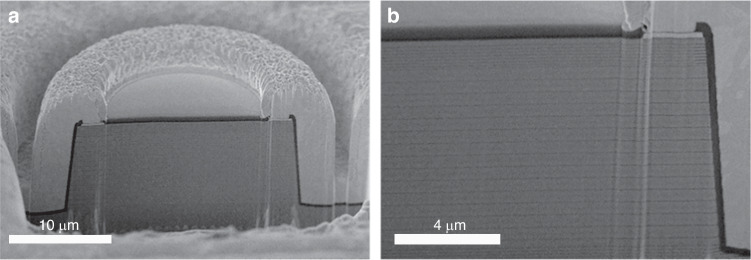
Cross-sectional SEM image of the 15-junction VCSEL. **a** the whole structure, the overall etching depth of the 15-junction VCSEL exceeded 9 μm. **b** Right-side view, the oxidation depth of the 15 oxide layers decreases sequentially from top to bottom

Figure 4 presents the cross-sectional scanning electron microscope (SEM) image of the 15-junction VCSEL, which was prepared by focused ion beam (FIB) etching. In this study, the oxidation aperture of all VCSELs was 10 μm. As shown in Fig. 4b, the overall etching depth of the 15-junction VCSEL exceeded 9 μm. Maintaining precise vertical etching was a highly challenging task, excessive etching angles would result in noticeable differences in the oxidation apertures between the topmost and bottommost oxide layers. Moreover, the epitaxial growth of high-junction VCSELs also posed technical challenges. Due to the accumulation of layer thickness, internal stress increased, which in turn affected the high-quality growth of multiple quantum wells (MQWs). Therefore, in the structural design of high-junction VCSELs, we considered using GaAsP quantum barriers for stress compensation.

To characterize the electrical and optical performance of individual VCSEL units with different junction numbers, we employed the light-current-voltage (L-I-V) test method driven by short pulses at room temperature. Detailed diagrams of the measurement Setup and measurement conditions can be found in Part Three of the supplementary materials. Short pulse testing was chosen to minimize the impact of thermal effects on the results, which is especially crucial for VCSELs with a high number of junctions. In laser radar applications based on the Time of Flight (TOF) scheme, a very high peak power is required to achieve longer detection distances. Therefore, the drive pulse width of multi-junction VCSELs is typically set to a few nanoseconds to significantly reduce the interference of thermal effects on device performance. For this experiment, we used a pulse width of 20 ns, a repetition rate of 50 kHz, and a duty cycle of 0.1%.

**Fig. 5:**
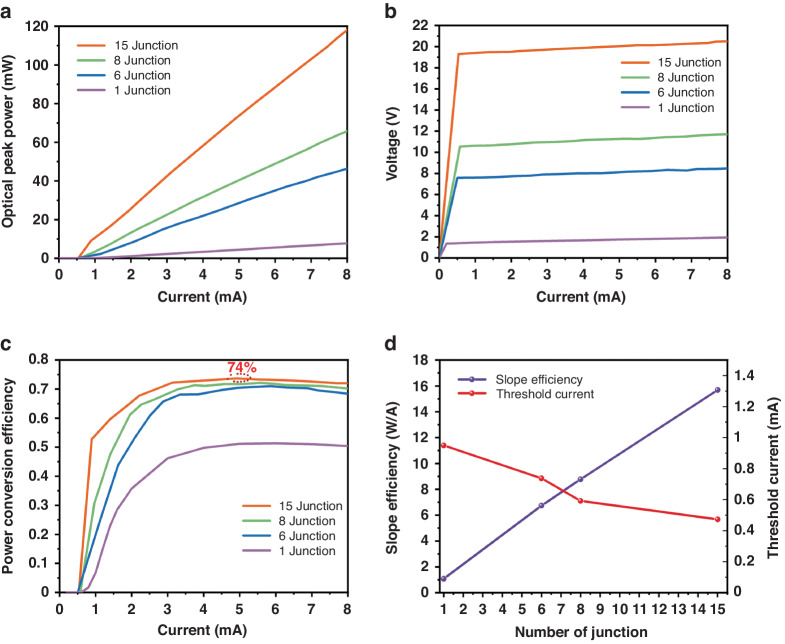
The optical and electrical characteristics of VCSELs with different numbers of junctions. **a**, **b** The L-I-V performance of VCSELs with different numbers of junctions, the 15-junction VCSEL surpasses 100 mW in peak power when the current is at 7 mA. **c** The PCE of VCSELs with different numbers of junctions, the maximum electro-optical conversion efficiency of the 15-junction VCSEL reached 74%. **d** The slope efficiency and threshold current of VCSELs with different numbers of junctions, the slope efficiency of the 15-junction VCSEL reached a significant 15.6 W/A, correspondingly, its differential quantum efficiency exceeded 1100%

The L-I-V performance of VCSELs with different numbers of junctions is shown in Fig. 5a and b. It can be observed that as the current increases, the output power grows approximately linearly, without thermal rollover phenomenon. Notably, the 15-junction VCSEL surpasses 100 mW in peak power when the current is at 7 mA. From Fig. 5b, it is evident that as the number of PN junctions increases, the turn-on voltage of the VCSEL also rises proportionally. For instance, the turn-on voltage for the 15-junction VCSEL has reached over 19 V. However, for applications involving short pulses and high modulation rates, this high voltage and low current characteristic is advantageous, as electrical signals with high voltage are more conducive to generating extremely short pulse widths and higher modulation rate signals compared to high current signals. From Fig. 5c, it can be observed that as the number of junctions increases, the peak PCE also rises, with the 15-junction VCSEL achieving a peak PCE of 74%. To the best of the author’s knowledge, this is the highest efficiency device in VCSELs to date. As shown in Fig. 5d, the slope efficiency of the 15-junction VCSEL reached a significant 15.6 W/A, correspondingly, its differential quantum efficiency exceeded 1100%. This differential quantum efficiency is the highest among all current semiconductor lasers. Further observing Fig. 5d, even though we designed a decreasing top DBR reflectivity with the increase in the number of junctions, the threshold current still shows a declining trend with the growth in junctions. In the future, we plan to further optimize the reflectivity of the top DBR. In addition, as the number of junctions increases, the growth rate of the peak PCE gradually slows down. The peak PCE of the 15-junction even falls below our numerical simulation results. This can be attributed to several reasons: As the number of junctions increases, we did not optimize the reflectivity during the epitaxial growth process to maintain consistency in the threshold current. An increase in the number of junctions implies an increase in the thickness of the epitaxial growth layer, leading to inconsistent quality between the top and bottom MQWs of the epitaxial growth. The internal quantum efficiency of VCSELs with more junctions is lower than that of single-junction VCSELs. This also explains why the equivalent single-junction slope efficiency of the 15-junction VCSEL is lower than the slope efficiency of the single-junction VCSEL although the reflectivity of the top DBR has been reduced.

**Fig. 6:**
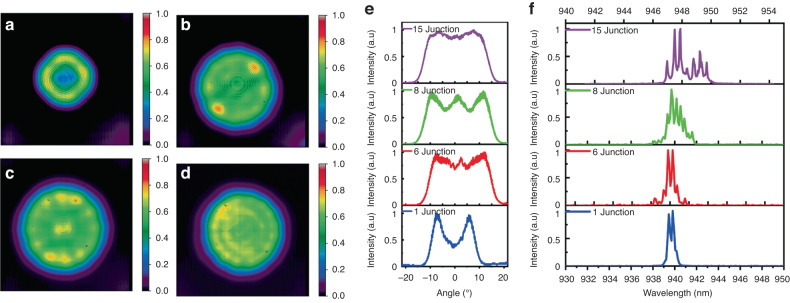
The far-field beam patterns and spectra of VCSELs with different numbers of junctions at a driving current of about 5 mA. **a**–**d** Respectively, represent the far-field beam patterns of 1-junction, 6-junction, 8-junction, and 15-junction VCSELs. The distribution of far-field beam clearly shows the existence of LP21, LP41, LP61, and LP81 modes, respectively. **e** Represents the normalized far-field divergence angle distribution of VCSELs with different numbers of junctions. **f** Represents the spectra of VCSELs with different numbers of junctions

Figure 6 shows the far-field beam patterns and spectra of VCSELs with different numbers of junctions at a driving current of about 5 mA. Figure 6a–d, respectively, represent the far-field beam patterns of 1-junction, 6-junction, 8-junction, and 15-junction VCSELs. Details of the testing setup can be found in the third section of the supplementary materials. For the tests, a pulse width of 20 ns and a repetition rate of 50 kHz were employed. Figure 6a–d distinctly shows that the quantity of modes increases with the rising number of junctions. At the same time, from the far-field beam pattern distribution, we can clearly see the LP21, LP41, LP61, and LP81 modes in 1-junction, 6-junction, 8-junction, and 15-junction, respectively. This is because, as the number of junctions increases, the corresponding number of oxidation layers also increases. According to the effective refractive index model^42^, the larger the number of oxidation layers, the lager is the difference in effective refractive index, resulting in stronger confinement capabilities for the optical field, allowing higher-order modes. Simultaneously, from the far-field divergence angle distribution shown in Fig. 6e, it is evident that, as the number of junctions increases, the far-field divergence angle also increases, with the divergence angle of the 6-junction VCSEL already at 28.8°. This confirms that the optical field confinement ability of multi-junction VCSELs is increased with the increased number of oxidation layers. However, a large divergence angle is disadvantageous for many application scenarios, hence, existing multi-junction VCSELs used in LiDAR typically reduce the number of oxide layers to decrease the divergence angle. In 2022, we reported that through the design of the oxide layers, the divergence angle in an 8-junction VCSEL was reduced to 18° for short pulse driving current^39^. Figure 6f shows the spectra of VCSELs with different numbers of junctions, clearly indicating an increase in the number of spectral peaks with an increase in the number of junctions, thereby signifying an increase in the number of modes.

## Discussion

**Fig. 7:**
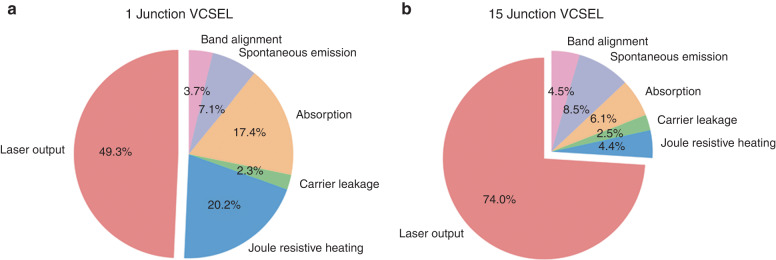
The energy consumption ratio of single-junction versus 15-junction VCSELs. **a** Single-junction VCSEL energy consumption ratio. **b** 15-junction VCSEL energy consumption ratio. Therefore, as the number of junctions increases, the corresponding proportion of joule heat and internal losses absorption decreases, leading to an improvement in PCE

To delve deeper into the PCE enhancement mechanism of the 15-junction VCSEL, we conducted a detailed power consumption analysis based on LIV curve data, referring to the methodology in reference^43^. The relevant results are shown in Fig. 7. The figure presents a comparison of the energy consumption distribution between single-junction and 15-junction VCSELs. Clearly, as the number of junctions increases, the dominant reduction in energy consumption is mainly attributed to the decrease in the proportion of Joule heat caused by resistance and internal losses due to free carrier absorption. This aligns with our previous theoretical analysis. As the number of junctions increases, the gain inside the cavity multiplies, but the device’s resistance and internal losses do not grow proportionally. In fact, due to the reduction in the number of layers of the top DBR, this is highly beneficial for reducing resistance and free carrier absorption. Therefore, as the number of junctions increases, the corresponding proportion of energy consumption decreases, leading to an improvement in PCE, which is precisely the advantage exhibited by multi-junction VCSELs.

**Fig. 8:**
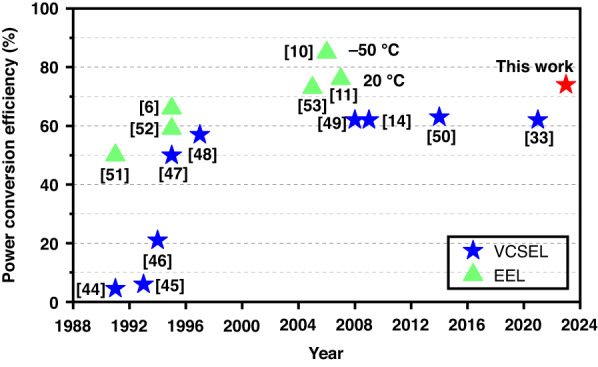
Summary of power conversion efficiency of semiconductor laser including representative VCSEL^14,33,44–50^ and EEL^6,10,11,51–53^. The power conversion efficiency of 74% was demonstrated using the 15-junction VCSEL, marking not only a significant improvement in VCSEL efficiency over the past two decades but also essentially reaching a level comparable to the highest power conversion efficiency of EELs under room temperature conditions

Figure 8 presents a summary of the power conversion efficiency of semiconductor lasers, including representative VCSELs and EELs. It can be observed that in 2008, EELs achieved a peak power conversion efficiency of 86% at low temperatures and 76% at room temperature. Over the past two decades, the efficiency of VCSELs has largely remained around 60%. Our use of multi-junction VCSELs has demonstrated the significant potential for improvement in VCSEL power conversion efficiency. An efficiency of 74% has been proven using a 15-junction VCSEL, which is very close to the highest room-temperature power conversion efficiency of EELs. This also represents a significant advancement in VCSEL laser sources.

We simulated the scaling characteristics of multi-junction VCSELs and compared to single-junction VCSELs. Numerical simulations reveal that the energy conversion efficiency of a 20-junction VCSEL under ambient temperature conditions could exceed 88%. This PCE value has already surpassed the highest PCE value reported under low-temperature operating conditions in experimental studies of EEL. Thus multi-junction VCSELs offer a novel method for improving the efficiency of semiconductor lasers. To validate this conclusion, we fabricated VCSEL devices with different numbers of junctions and conducted a detailed analysis of their energy dissipation characteristics. The analysis indicates that as the number of junctions increases, the key to the PCE improvement of multi-junction VCSELs lies in the fact that while the gain increases multiplicatively, the resistance and internal losses do not increase proportionally, reducing the proportion of Joule heat and internal loss absorption in device energy consumption. Encouragingly, experimental data shows that the 15-junction VCSEL, driven by nanosecond pulses, achieved an energy conversion efficiency of 74% in a room temperature environment. To our knowledge, this efficiency is the highest reported in the VCSEL field to date. The slope efficiency of the 15-junction VCSEL is 15.6 W/A, and its corresponding differential quantum efficiency exceeds 1100%. To our knowledge, this differential quantum efficiency is the highest reported in the semiconductor laser field to date. At the same time, for high-junction VCSELs to achieve the same power, they operate at low current and high voltage conditions, which also provides conditions for ultra-short pulse and high modulation rate applications. We also plan to explore the expansion of high-efficiency, high-power multi-junction VCSEL applications in the field of communications in the future. This study not only provides valuable theoretical and experimental evidence for the further optimization and application of VCSELs but also it will provide valuable references for the further development and application of high PCE semiconductors laser and have a significant impact on photonics and laser physics.

## Materials and methods

### Materials grown

The top-emitting 940 nm multi-junction VCSEL was based on an n-type GaAs substrate and was grown using metal-organic chemical vapor deposition (MOCVD) technology. The bottom DBR consisted of 41 pairs of N-doped Al_0.07_Ga_0.93_As/Al_0.9_Ga_0.1_As. For the 1-junction, 6-junction, 8-junction, and 15-junction VCSELs, their top DBRs were made up of 19 pairs, 11 pairs, 9 pairs, and 7 pairs of P-doped Al_0.07_Ga_0.93_As/Al_0.9_Ga_0.1_As, respectively. Each cascaded active region includes three pairs of quantum wells and barriers of In_0.14_Ga_0.86_As and GaAs_0.88_P_0.12_, with thicknesses of 7 nm and 6 nm, respectively. The optical thickness of the cavity length for each cascaded active region was uniformly 2λ. The oxidation layer was composed of 20 nm Al_0.98_Ga_0.02_As and was located at the first node of standing wave field above the quantum well. The tunnel junction is made up of 15 nm thick P^++^ and N^++^ GaAs, where the doping concentration for P-type is 1e20 cm^−2^ and for N-type is 1e19 cm^−2^.

### Fabrication

The manufacturing process is as follows: Firstly, P-type contact metal is deposited on the top of the P^+^ contact layer on the p-side, forming a ring-shaped electrode structure. Then, dry etching is performed using chlorine-based gas, exposing the high Al oxidation layer. Afterward, through a high-temperature and high-humidity oxidation furnace process, oxidation apertures are formed with a diameter of 10 μm each. To isolate the device, we use PECVD technology to deposit Si_3_N_4_ as a passivation layer. Subsequently, through grinding and polishing processes, the substrate thickness is reduced to 80 μm. The final step is to deposit gold on the substrate to form an n-type electrode.

### Measurement

To ensure the short pulse signal is not distorted, each test sample was directly mounted on the test circuit board and the board was directly connected to the short pulse drive power source (AVTEK, AV-1010-B) to minimize the parasitic impedance of the current loop. The pulse width for LIV testing is 20 ns, with a frequency of 50 kHz and a duty cycle of 0.1%. In addition, we used an oscilloscope (Keysight EXR104A) to monitor the voltage signal at both ends of the device in real-time, while the optical power was measured by a power meter (Ophir 3A-IS). Spectral testing using a spectrometer (Ocean, HR4000). Detailed testing methods can be found in Part Three of the supplementary materials.

### Supplementary information

supply mateial V5 20240123modify
