# Supplementary material for: Multi-junction cascaded vertical-cavity surface-emitting laser with a high power conversion efficiency of 74%
Source: Light Sci Appl. 2024 Feb 28;13:60. doi: 10.1038/s41377-024-01403-7 (PMC10899599; doi:10.1038/s41377-024-01403-7)
Supplement: Supplementary file 1 — supply mateial V5 20240123modify [file 41377_2024_1403_MOESM1_ESM.docx]

**Supplementary information**

Multi-junction cascaded vertical-cavity surface-emitting laser with a high power conversion efficiency of 74%

Yao Xiao^1^, Jun Wang^1,2, *^, Heng Liu ^2^, Pei Miao^1,2^, Yudan Gou^1^, Zhicheng Zhang^1^, Guoliang Deng^1^, and Shouhuan Zhou^1^

*^1^College of Electronics and Information Engineering, Sichuan University, Chengdu 610064, China*

^2^Suzhou Everbright Photonics Co., Ltd, Suzhou 215163, China

Emails: wjdz@scu.edu.cn;

1. **Multi-junction VCSEL's scaling behavior theory**


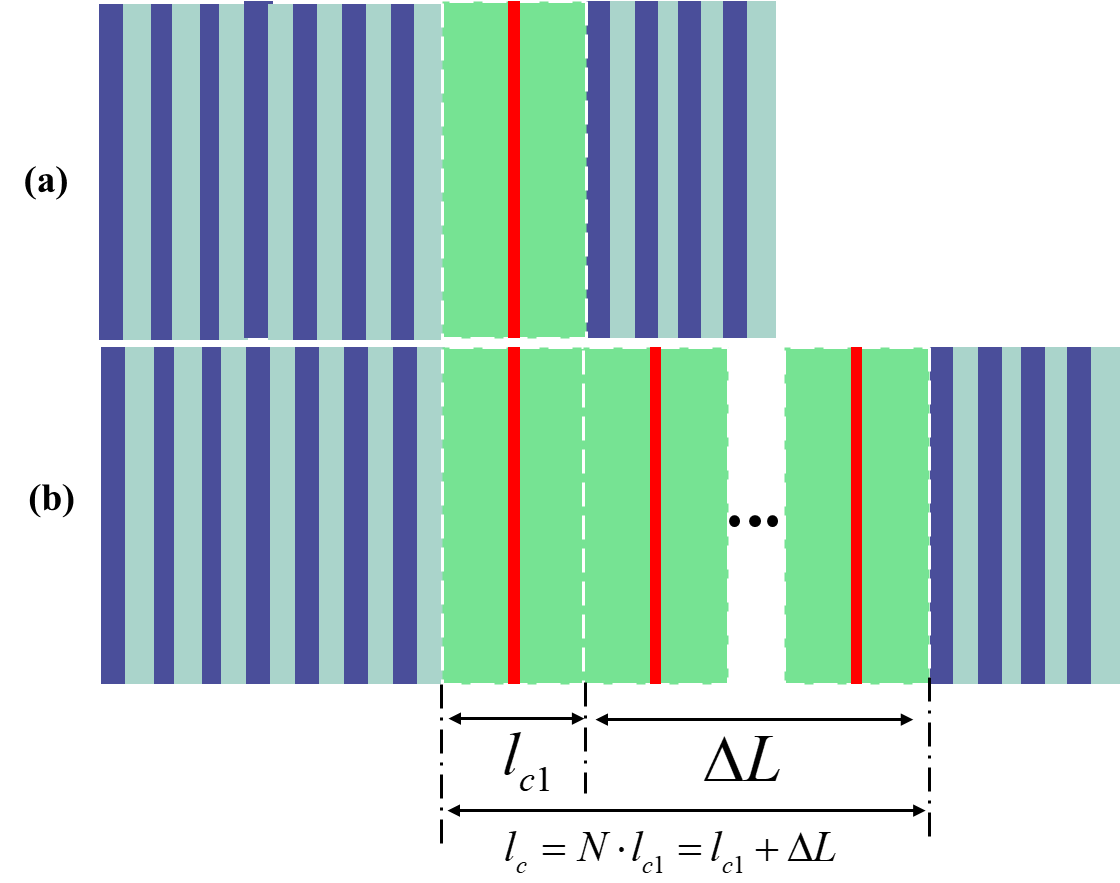


**Supplementary Figure S1. Schematic diagrams of the single-junction VCSEL and multi-junction VCSEL structures**

Based on the fundamental performance of the single-junction VCSEL, we derive the scaling behavior of the electro-optical performance for multi-junction. As shown in Figure S1: Schematic diagrams of the single-junction VCSEL and multi-junction VCSEL structures are presented. The single-junction VCSEL mainly consists of the top and bottom DBRs and the active region in the middle, which contains the quantum well gain layer and the oxidation confinement layer. The DBRs form mirrors at the top and bottom, thereby creating a resonator. For top-emitting VCSELs, the bottom DBR serves as a high-reflectance mirror. The active region of the multi-junction VCSEL has a periodic repeating structure, allowing for an extended cavity length. The designed optical thickness of a single active region is 2λ. Therefore, the thickness of the single-junction VCSEL cavity is very small, classifying it as a microcavity laser. Its longitudinal mode spacing is very large, allowing for single longitudinal mode output. This is also a crucial reason why it has found widespread applications in sensing (such as atomic clocks) and optical communication. However, as the number of cascades in the multi-junction VCSEL increases, the spacing between its longitudinal modes gradually decreases. The specific calculation formula is as follows^1^:

$\Delta\lambda_{1}\approx\frac{\lambda^{2}}{2L_{\text{eff}}\left\langle\bar{n}_{gr}\right\rangle}$ (1)

It can be inferred that the longitudinal mode spacing of the 15-junction VCSEL is approximately 15nm. Therefore, the secondary mode spacing on both sides of the main mode is 30nm. The temperature drift coefficient of the VCSEL cavity film is 0.07 nm/℃. The temperature drift coefficient for the gain peak of the InGaAs quantum well is 0.3 nm/℃. The working temperature range to ensure no mode hopping is 130℃. Typically, sensors and communications require devices to operate from -40℃ to 90℃. Therefore, the 15-junction VCSEL is the limit for most current applications. Thus, the maximum number of junctions studied in this paper is 15. This is also the highest cascaded junction number reported in multi-junction VCSELs.

For a VCSEL, the threshold condition for a laser is as follows^2^:

$G_{1}=\alpha_{1i}+\alpha_{1m}$ (2)

$\Gamma\text{g}_{th}=\alpha+\frac{1}{l_{c1}}\ln\frac{1}{\sqrt{R_{b}R_{t}}}$ (3)

Where G_1_ is the gain of the single-junction VCSEL, α_1i_ is the internal loss of the single-junction VCSEL, α_1m_ is the mirror loss of the single-junction VCSEL, Γ is the optical confinement factor, α is the internal loss, R_b_ and R_t_ are the reflectivity’s of the bottom and top, respectively, Here, the calculated bottom reflectivity is set to 100%. Figure S1(a) Schematic diagram of the single-junction VCSEL structure, (b) Schematic diagram of the multi-junction VCSEL structure. For the multi-junction VCSEL, the threshold conditions are as follows:

$$G_{N}=\alpha_{Ni}+\alpha_{Nm}\text{ （}4\text{）}$$

$$G_{N}=\alpha_{1i}+\alpha_{1m}+\Delta\alpha_{i}+\Delta\alpha_{m}\text{ （}5\text{）}$$

$$\begin{matrix} \Delta\alpha_{i}=\alpha_{Ni}-\alpha_{1i},\Delta\alpha_{m}=\alpha_{Nm}-\alpha_{1m}\text{ （}6\text{）} \\ G_{N}=G_{1}+\Delta\alpha_{i}+\Delta\alpha_{m}\text{ （}7\text{）} \end{matrix}$$

Where G_N_ is the gain of the multi-junction VCSEL, $\Delta\alpha_{i}$Represents the additional internal loss in the cavity, $\Delta\alpha_{m}$Represents the additional mirror loss, For the multi-junction VCSEL, the reflectivity is defined as: $R_{b}R^{'}_{t}=\zeta R_{b}R_{t}$. Therefore, from equation (7), the threshold gain of the multi-junction VCSEL is as follows:

$$g_{n}=\frac{g_{1}}{N}+\frac{\alpha_{i}\Delta l_{c}}{\Gamma l_{a1}N}-\frac{\ln\sqrt{\zeta}}{\Gamma l_{a1}N}\text{ （}8\text{）}$$

For the single-junction VCSEL, the differential gain is as follows:

$$\eta_{d1}=\eta_{i1}\frac{\alpha_{m}}{\Gamma g_{th}}=\eta_{i}\frac{\alpha_{m}}{\alpha_{m}+\alpha_{i}}\text{ （}9\text{）}$$

Where $\eta_{i}$represents the current injection efficiency,

Assuming that the injection efficiency of all active regions in the cascade is consistent, the differential gain of the multi-junction VCSEL is as follows:

$$\eta_{dN}=N\eta_{i1}\frac{\alpha_{1m}+\Delta\alpha_{m}}{\alpha_{1m}+\Delta\alpha_{m}+\alpha_{1i}+\Delta\alpha_{i}}\text{ （}10\text{）}$$

According to equation 8, the threshold currents for both single-junction and multi-junction are:

$J_{1}=J_{tr}\exp(\frac{g_{1}}{g_{0}})$ （11）

$J_{N}=J_{tr}\exp(\frac{g_{N}}{g_{0}})$ (12)

$J_{N}=J_{1}\exp\left[\frac{1}{g_{0}N}\left(g_{1}(1-N)+\frac{\alpha_{i}\Delta l_{c}}{\Gamma l_{a}}-\frac{\ln\sqrt{\zeta}}{\Gamma l_{a}}\right)\right]$ (13)

Where g_0_​ is the gain coefficient.

The voltage for single-junction and multi-junction VCSELs is as follows:$V_{1}=V_{DBR}+V_{d}$ (14)

$V_{N}=V_{DBR}+NV_{d}+(N-1)V_{TJ}$ (15)

$V_{N}=V_{1}+(N-1)(V_{d}+V_{TJ})$ ）（16）

Therefore, the power conversion efficiency for single-junction and multi-junction can be obtained as follows:

$PCE=\frac{P_{\text{laser}}}{VI}=\frac{(I-I_{th})slope}{IV_{on}+IR},slope=\eta_{dN}\frac{\text{h}\nu}{e}$ (14)

Here, we discuss two scenarios. First, with a fixed reflectivity of the output mirror, second, by adjusting the reflectivity of the output mirror to maintain a consistent threshold, the power conversion efficiency is as follows:

$\eta{}_{PCEN}=\eta_{PCE1}\frac{I-I_{th1}\exp\left\{\frac{g_{th}\text{（}1-N_{a}\text{）}}{g_{0}N_{a}}\right\}}{I-I_{th1}}\frac{\ln\sqrt{R_{b}R_{t}}}{\ln\sqrt{R_{b}R_{t}}+\eta_{d1}(1-\alpha\Delta L)/\eta_{i1}}\frac{N_{a}(V_{1}+IR)}{N_{a}V_{1}+IR_{N}}\left|\zeta=1\right.$ (14)

$\eta{}_{PCEN}=\eta_{PCE1}\frac{\ln\sqrt{\zeta R_{b}R_{t}}}{\ln\sqrt{R_{b}R_{t}}+\eta_{d1}/\eta_{i}\ln\sqrt{\zeta}}\frac{N_{a}(V_{1}+IR)}{N_{a}V_{1}+IR_{N}}\left|g_{1}=\right.g_{N}$ (15)

Where, $g_{1}=g_{N}$，According to equation8, it can be derived that, with the threshold kept constant, the proportion of reflectivity change is:

$\ln\sqrt{\zeta}=\exp\left\{\Gamma l_{a}g_{1}(1-N_{a}+\alpha\Delta L\right\}$(16)

**Supplementary Tabel 1 Parameters used for simulations**

| **Symbol** | **Parameter** | **Value** |
| --- | --- | --- |
| g_0_ | Gain Coefficient | 1600 cm^-1^ |
| ΔL | Cavity Length | (N_a_-1)*0.6 μm |
| N_a_ | Number of junctions | 6,8,15 |
| g_th_ | Single Junction Threshold Gain | 2200 cm^-1^ |
| η_i_ | Internal Quantum Efficiency | 0.95 |
| Γ | Gain Enhancement Factor | 1.8 |
| R_b_ | Bottom Reflectivity | 0.999 |
| R_t_ | Single Junction Bottom Reflectivity | 0.995 |
| I_th1_ | Single Junction Threshold Current | 0.8 mA |

1. Device Fabrication

Using the standard VCSEL fabrication process, the flowchart is as follows:


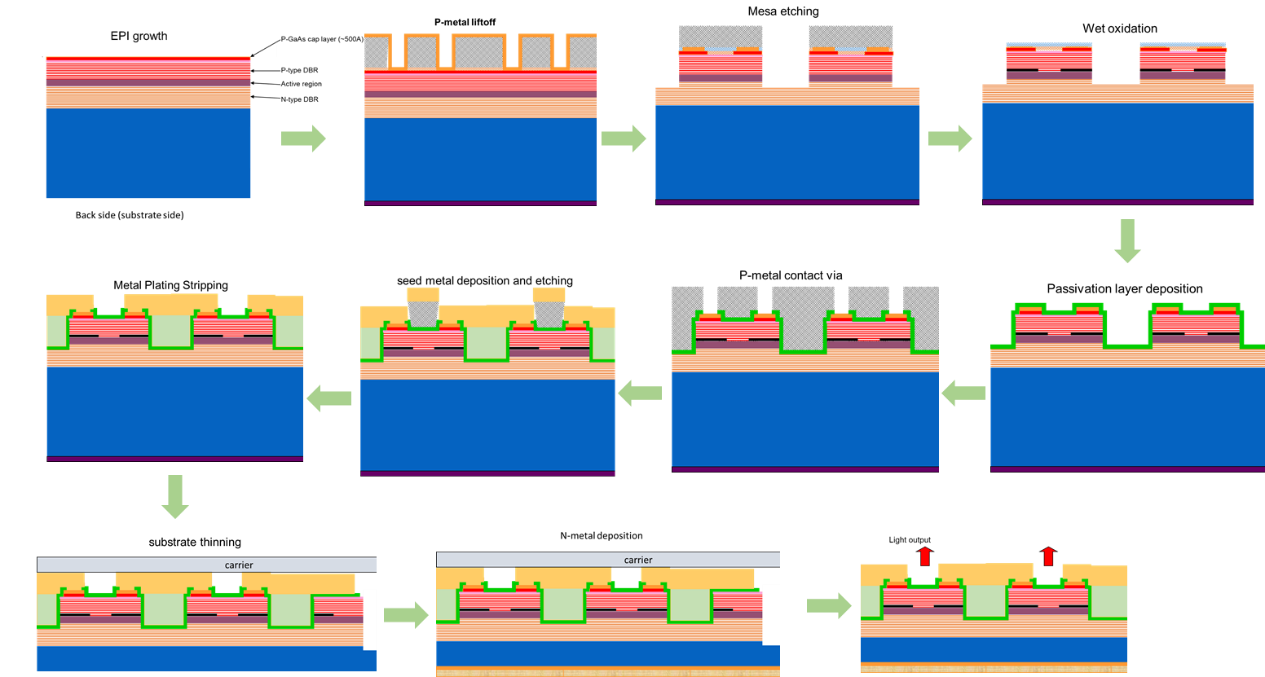


**Supplementary Figure S2. Process flowchart**

1. Measurement Setup

The setup for light-current-voltage (L-I-V) testing is depicted in Supplementary Figure S3(a). Our testing methods adhere to the standard short-pulse testing protocols in our field, as referenced in our literature.^3-5^ The driving power source for the VCSEL is a voltage-driven short pulse power source (AVTEK, AV-1010-B). The pulse width for LIV testing is 20 ns, with a frequency of 50 kHz and a duty cycle of 0.1%. VCSEL is serially connected to a sampling resistor with a resistance of 50 ohms. The current in the loop is obtained by measuring the voltage across the sampling resistor using an oscilloscope (Keysight EXR104A), and this current is the driving current for the VCSEL. Simultaneously, the voltage across the positive and negative terminals of the VCSEL is directly measured using an oscilloscope. The power of the VCSEL is collected using a power meter(Ophir 3A-IS), with the measured power being the average power. The peak power is obtained by dividing the average power by the duty cycle. Supplementary Figure S4 presents the sampling resistor, voltage signals at both ends of the 15 junction VCSEL, and the laser pulse signals measured by a high-speed photodetector (IC Haus, IC212NST) during the test process. As depicted in Supplementary Figure S4(a), the voltage signal at the ends of the sampling resistor indicates that the tested pulse signal's FWHM is about 20 ns, aligning with the predetermined 20 ns, and closely resembles a square wave signal. The voltage signal at both ends of the 15 junction VCSEL, as shown in Supplementary Figure S4(b), has a pulse waveform consistent with that across the sampling resistor. The laser, possessing capacitive properties, continues to exhibit signal oscillations even after the drive is halted. The laser pulse signals were tested using a high-speed photodetector, as illustrated in Supplementary Figure S4(c) and (d). The FWHM of the laser pulses is 20 ns, and the shape of the light pulse is essentially identical to the electrical signal pulse from the driving source. As shown in Figure S4(d), the repetition frequency of the laser pulse signal is 50 kHz, matching the set value, and the peak values of the laser pulse sequences are very stable. The setup for far field testing is depicted in Supplementary Figure S3(b). The distance from the CCD camera (Visiondatum, Mars25MP-43Tgm) to the surface of the chip is 29.3 mm.

**
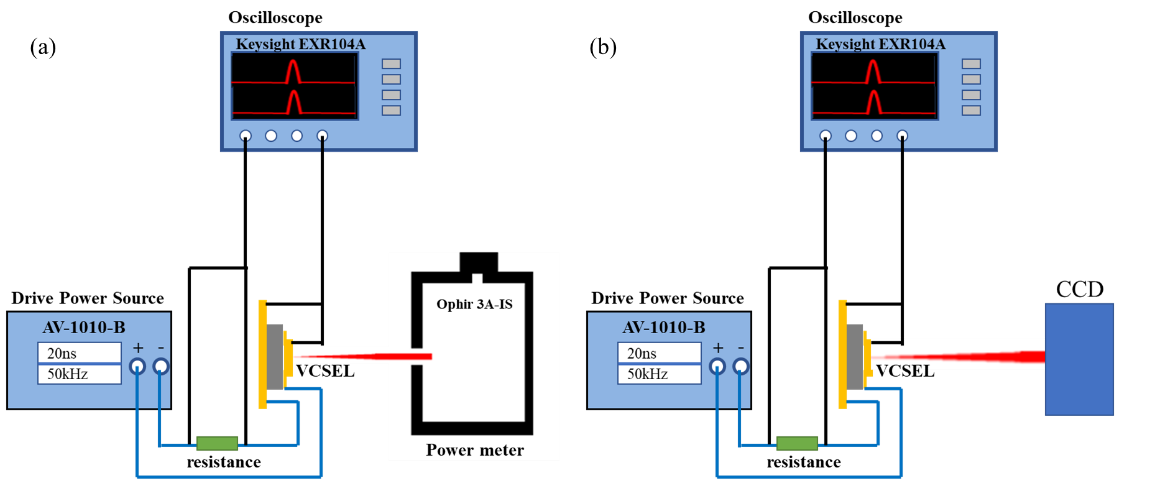
**

**Supplementary Figure S3. (a) Measurement setup for light-current-voltage. (b) Measurement setup for far field**

**
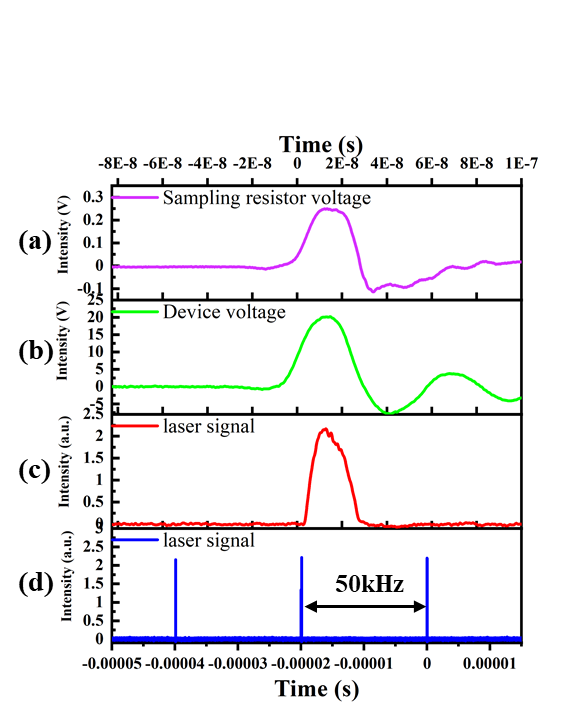
**

**Supplementary Figure S4. the sampling resistor, voltage signals at both ends of the 15 junction VCSEL, and the laser pulse signals (a) voltage signals at both ends of the sampling resistor, (b) voltage signals at both ends of the 15 junction VCSEL, (c) and (d) the laser pulse signals.**

Reference:

1 Michalzik, R. in *VCSELs: fundamentals, technology and applications of vertical-cavity surface-emitting lasers* 19-75 (Springer, 2012).

2 Coldren, L. A. *et al.* *Diode lasers and photonic integrated circuits*. (John Wiley & Sons, 2012).

3 Kang, D. *et al.* Compliant, Heterogeneously Integrated GaAs Micro-VCSELs towards Wearable and Implantable Integrated Optoelectronics Platforms. **2**, 373-381 (2014).

4 Pandey, A. *et al.* High-efficiency AlGaN/GaN/AlGaN tunnel junction ultraviolet light-emitting diodes. *Photonics Res.* **8**, 331-337 (2020).

5 Wang, Z. *et al.* Ultra-low threshold lasing through phase front engineering via a metallic circular aperture. *Nat. Commun.* **13**, 230 (2022).
